# Banded versus non-banded sleeve gastrectomy: A systematic review and meta-analysis

**DOI:** 10.1097/MD.0000000000032982

**Published:** 2023-04-14

**Authors:** Mohamed Ali Chaouch, Wah Yang, Amine Gouader, Bassem Krimi, Adriano Carneiro da Costa, Guillaume Pourcher, Hani Oweira

**Affiliations:** a Department of Visceral and Digestive Surgery, Fattouma Bourguiba Hospital, University of Monastir, Monastir, Tunisia; b Department of Digestive, Metabolic, and Oncologic Surgery, Institut Mutualiste of Montsouris, Paris, France; c Department of Metabolic and Bariatric Surgery, The First Affiliated Hospital of Jinan University, Guangzhou, China; d Department of Visceral and Obesity Surgery, Perpignan Hospital Center, Perpignan, France; e Department of Surgery, Universitäts medizin Mannheim, Heidelberg University, Mannheim, Germany.

**Keywords:** banded sleeve gastrectomy, bariatric surgery: sleeve gastrectomy, complication, non-banded sleeve gastrectomy, weight loss

## Abstract

Laparoscopic banded sleeve gastrectomy (LBSG) has been compared to laparoscopic sleeve gastrectomy (LSG) in terms of anthropometric results and postoperative complications, which are controversial. This systematic review and meta-analysis aimed to compare the safety and efficacy of LBSG and LSG.

**Methods:**

We performed a systematic review with meta-analysis according to preferred reporting items for systematic review and meta-analysis 2020 and assessing the methodological quality of systematic review 2 guidelines. We included studies that systematically searched electronic databases and compared LBSG with LSG conducted until August 10, 2021.

**Results:**

The literature search yielded 8 comparative studies. Seven hundred forty-three patients were included: 352 in the LBSG group and 391 in the LSG group. LBSG group allowed greater anthropometric parameters (body mass index [BMI] after 1 year (mean difference [MD] = −3.18; 95% CI [−5.45, −0.92], *P* = .006), %EWL after 1 year (MD = 8.02; 95% CI [1.22, 14.81], *P* = .02), and %EWL after 3 years (MD = 10.60; 95% CI [5.60, 15.69], *P* < .001) and similar results with LSG group in terms of operative time (MD = 1.23; 95% CI [−4.71, 7.17], *P* = .69), food intolerance (OR = 1.72; 95% CI [0.84, 3.49], *P* = .14), postoperative vomiting (OR = 2.10; 95% CI [0.69, 6.35], *P* = .19), and De novo GERD (OR = 0.65; 95% CI [0.34, 1.26], *P* = .2). Nevertheless, major postoperative complications did not differ between the 2 groups.

**Conclusions:**

This systematic review and meta-analysis comparing LBSG and LSG concluded that banding sleeve gastrectomy (SG) may ensure a lower BMI and %EWL after 1 year of follow-up, and a significant reduction in %EWL after 3 years of follow-up. There is no evidence to support LBSG in vomiting, de novo GERD, food intolerance, or operative time.

## 1. Introduction

The indications for bariatric surgery are consensual, but the best procedure is controversial. Since the advent of laparoscopy, this surgery has sustained significant progress.^[1]^ Laparoscopic sleeve gastrectomy (LSG) is the most widely performed procedure.^[2]^ Owing to its encouraging early postoperative results, it was converted to a stand-alone procedure. However, a significant remaining problem is the long-term weight regain of 28% and the requirement for revision surgery of 19.9%.^[3]^ This could be due to surgeon-dependent and patient-dependent factors, remnant gastric tube dilation, and loss of some restrictive aspects of the sleeve.^[4,5]^ Consequently, some surgeons have proposed modifications to surgical procedures and have used a band around the gastric tube to improve postoperative outcomes, as was used for banded Roux-en-Y gastric bypass (GBP) to stabilize the size of the gastric pouch.^[6]^ Regarding the more significant results in long-term weight loss in the banded GBP group,^[6]^ similar studies have been performed in laparoscopic banded sleeve gastrectomy (LBSG).^[7]^ This strategy seems to be logical. However, these results remain controversial. This systematic review and meta-analysis examined the anthropometric and postoperative outcomes after LBSG and LSG.

## 2. Methods

This systematic review and meta-analysis was performed according to the Preferred Reporting Items for Systematic Review and Meta-analysis guidelines 2020^[8]^ and assessing the methodological quality of systematic reviews 2 guidelines.^[9]^ The protocol was registered in PROSPERO with the ID CRD42021279242.

### 2.1. Electronics searches

We performed an electronic investigation of relevant literature published during the last 2 decades on August 10, 2021. We did not use language restrictions. We searched the Cochrane Library Controlled Trials Registry and systematic review database, Embase, National Institutes of Health PubMed/MEDLINE, and Google Scholar. The following search terms were used: “banded,” “non-banded,” “banding,” “silastic band,” “sleeve gastrectomy," and “bariatric surgery.” The titles and abstracts of the studies were screened for relevancy. We manually checked the reference lists of the relevant reviews for additional citations.

### 2.2. Inclusion and exclusion criteria

We retained randomized controlled trial (RCTs) and high-quality controlled clinical trials (CCTs) comparing the efficacy of banded and non-banded sleeve gastrectomy (SG) in adults (>18-year-old) with clinically severe obesity (body mass index [BMI] > 35 kg/m^2^). Only articles published in peer-reviewed journals were included in this study. Data from non-comparative studies, review articles, editorials letters, abstracts only, comments, and case series (fewer than 10 cases) were excluded.

We included RCTs and CCTs only because trials with this design would provide the most substantial evidence to answer our clinical question related to the efficacy of the investigated interventions.

### 2.3. Outcomes measures

The primary outcome of this meta-analysis was anthropometric parameter changes after banded versus non-banded SG (BMI and %EWL after 1 year, results after 3 years and 5 years of follow-up).

The secondary outcomes were operative time, postoperative vomiting, incidence of food intolerance, new-onset reflux, early operative revision, and resolution of comorbidities. We assessed the postoperative complications during the follow-up period.

### 2.4. Study selection

Two authors independently reviewed all abstracts. We retained all studies accompanied by the full text that met the inclusion criteria. After consulting a third review team member, disagreements were resolved through discussions.

### 2.5. Data extraction

Two authors extracted the data independently, and the senior authors settled the disparities after discussion included studies were fully matched for the first author name, year of publication, type of study, country, sample size (banded group vs non-banded group), study period, mean age, sex, preoperative BMI, and comorbidities.

### 2.6. Missing data

We contacted authors by e-mail regarding the occurrence of unclear bias domains or missing primary outcome information of our meta-analysis. If the data were not reported numerically, they were extracted from the figures.

### 2.7. Assessment of studies quality and risk of bias assessment

Two authors independently appraised all studies that met the selection criteria. For the retained CCTs, we used the risk of bias in nonrandomized studies (ROBINS-I).^[10]^ We evaluated bias in 7 distinct domains (bias due to confounding, bias in selection of participants into the study, bias in classification of interventions, bias due to deviations from intended interventions, bias due to missing data, bias in measurement of outcomes, bias in selection of the reported result, and overall bias). Within each domain, 1 or more signaling questions led to judgments of “low risk of bias,” “moderate risk of bias,” “serious risk of bias,” “critical risk of bias,” or “no information.” We used the Cochrane tool for bias assessment to assess the risk of bias in randomized trials (RoB2).^[11]^ We evaluated bias in 5 distinct domains (randomization process, deviations from intended interventions, bias in the measurement of outcome, bias to missing outcome data, bias in selecting the reported results, and overall bias). Within each domain, 1 or more signaling questions led to judgments of “low risk of bias,” “some concerns,” or “high risk of bias” high risk of bias.

### 2.8. Handling continuous data

Continuous data were analyzed using the Review Manager 5.3.5 statistical package from Cochrane collaboration for meta-analysis.^[12]^ When the mean and standard deviation (SD) were not reported, they were estimated from the provided interquartile range (IR) and median based on the formula described by Hozo et al.^[13]^ If the sample size was > 25, the mean was equal to the median. In addition, the SD was calculated as IR/4 for a sample size <70 patients and IR/7 for a sample size >70 patients.

### 2.9. Assessment of heterogeneity

We used the Cochrane Chi^2^ test (Q-test), *I*^2^ statistic, and variance TAU^2^ to estimate the degree of heterogeneity.^[14]^ Funnel plots were used to identify studies responsible for heterogeneity. A subgroup analysis was performed when all the included studies reported outcomes.

### 2.10. Summary of findings

Two authors independently assessed the evidence of the primary outcomes. Grading of Recommendations Assessment, Development, and Evaluation.^[15]^ We considered the study limitations in terms of the constancy of effect, imprecision, indirectness, and publication bias. We assessed the certainty of the evidence as high, moderate, low, or very low. If appropriate, we considered the following criteria for upgrading the evidence: large effect, dose-response gradient, and plausible confounding effect. We used the methods and recommendations described in sections 8.5 and 8.7 and chapters 11 and 12 of the Cochrane Handbook for Systematic Reviews of Interventions. We used GRADEpro GDT software to prepare a summary of the findings tables. We explained the reasons for downgrading or upgrading the included studies using footnotes and comments.

### 2.11. Evaluation of effect size

We used the RevMan 5.4 statistical package from the Cochrane collaboration for meta-analysis.^[12]^ We selected the mean difference (MD) as an effective measure of continuous data. Odds ratios (OR) with 95% confidence intervals (95% CI) were calculated for dichotomous variables. A random effects model was used. The threshold of significance was set at *P* < .05.

## 3. Results

### 3.1. Literature review

**Figure 1.**
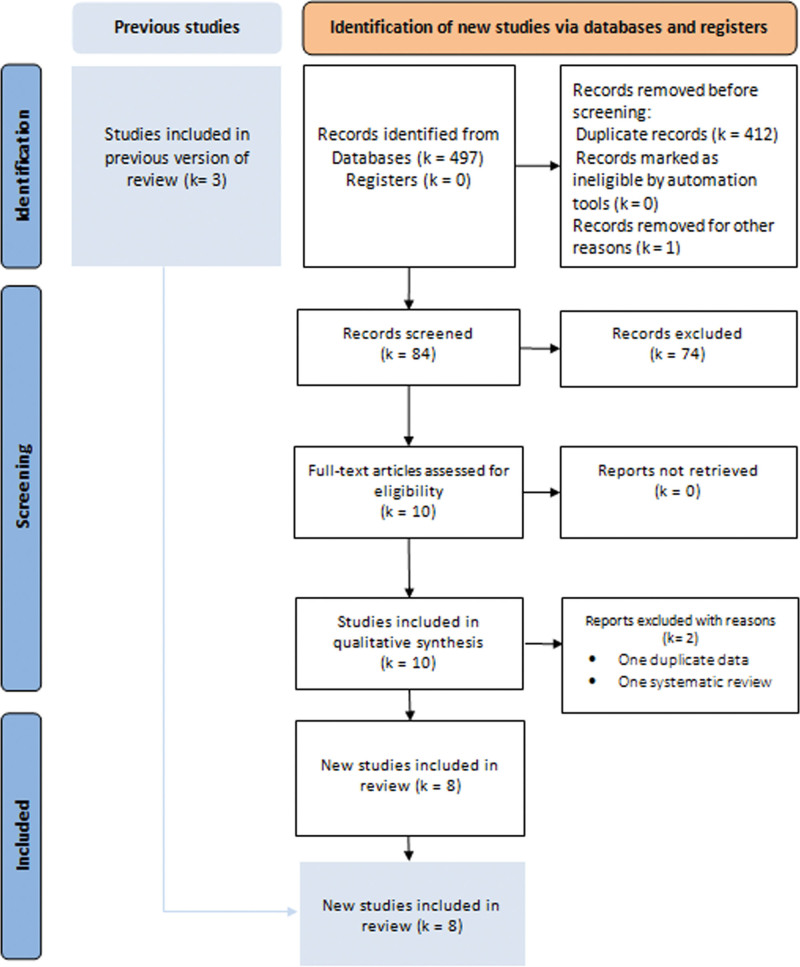
PRISMA 2020 flow diagram for search strategy, literature screening, and study selection. PRISMA = preferred reporting items for systematic review and meta-analysis.

The literature search yielded 11 eligible studies. We retained 8 comparative studies (Fig. 1)^[16–23]^: 3 RCTs,^[21–23]^ 4 CCTs,^[16,18–20]^ and 1 prospective non-randomized clinical trial.^[17]^ Two studies were excluded: 1 study presented duplicated data from the retained studies,^[17]^ and 1 meta-analysis of RCTs of banded and non-banded GBP.^[6]^ Seven hundred forty-three patients were included:352 in the LBSG group and 391 in the LSG group. The demographic data of the included patients are presented in Table 1.

**Table 1:** Demographic and data of the retained studies.

First author	Journal	Year of publication	Type of publication	Country	Study period	LBSG	LSG	Band used	Band closure circumference (cm)	Band location/OGJ (cm)	Gastric tube	Age (yr)		Gender (female/male)		Preoperative BMI (kg/m^2^)		Follow-up (mo)
												LBSG	LSG	LBSG	LSG	LBSG	LSG	
Bhandari	*Surgery for Obesity and Related Diseases*	2019	PNRT	India	2012–2017	68	152	GaBP Ring Autolock	7.5	4–5	36 to 38 F	40.5 ± 12.6	40.4 ± 13.2	21/37	81/71	44.6 ± 8.3	45.0 ± 8.4	60
Fink 2017	*Obesity Surgery*	2017	CCT	Germany	January 2012 to October 2014	42	42	MiniMizer ring	7.5	4–6	35 F	40.05 ± 12.08	42.21 ± 13.49	30/12	28/14	54.93 ± 7.42	53.46 ± 6.69	36
Fink 2020	*Annals of Surgery*	2020	RCT	Germany	January 2015 to August 2019	47	47	MiniMizer ring	7.5	4	35 F	43.3	40.9	65%	69%	51 (49.1–53)	50.7 (48.8–52.6)	36
Gentileschi	*Journal of Obesity*	2020	RCT	Italy	January 2014 to January 2015	25	25	GaBP Ring Autolock	7–7.5	4	36 F	47.3 ± 6.58	43.7 ± 9.8	14/11	16/9	45.95 ± 5.85	47.3 ± 6.58	48
Karcz	*Obesity Surgery*	2014	CCT	Germany	January 2012 to August 2012	25	25	MiniMizer ring	6.5	6.5	35 F	42.6	43.6	18/7	17/8	56.1 ± 7.2	57.0 ± 6.3	12
Lemmens	*OBESITY surgery*	2018	CCT	Belgium - USA	May 2010 to July 2017	96	51	MiniMizer ring	6.5–7.5	4–5	40 F	47.9 ± 12.2	54.8 ± 14.1	36/60	29/22	43.7 ± 7.3	44.9 ± 7	60
Soliman	*Bariatric Surgical Practice and Patient Care*	2015	CCT	United Arab Emirates—Egypt	Jun 2011 to July 2012	24	24	Double layer mesh (Gortex mesh)	6.5–7	6	36 to 38 F	26 (18–62)	28 (19–61)	6/18	14/12	45.6 ± 4.83	−52.73 ± 5.85	12
Tognoni	*Gastroenterology Research and Practice*	2016	RCT	Italy	January 2014 to January 2015	25	25	GaBP Ring Autolock	7–7.5	4	36 F	45.7 ± 12.7	43.7 ± 9.8	16/9	16/9	44.95 ± 5.85	47.3 ± 6.58	12

CCT = clinical controlled trial, F = French, GERD = pre-operative gastroesophageal reflux disease, LBSG = laparoscopic banded sleeve gastrectomy, LSG = laparoscopic sleeve gastrectomy, OGJ = gastroesophageal junction, PNRT = prospective non-randomized trial, RCT = randomized controlled trial.

### 3.2. Anthropometrics parameters

**Figure 2.**
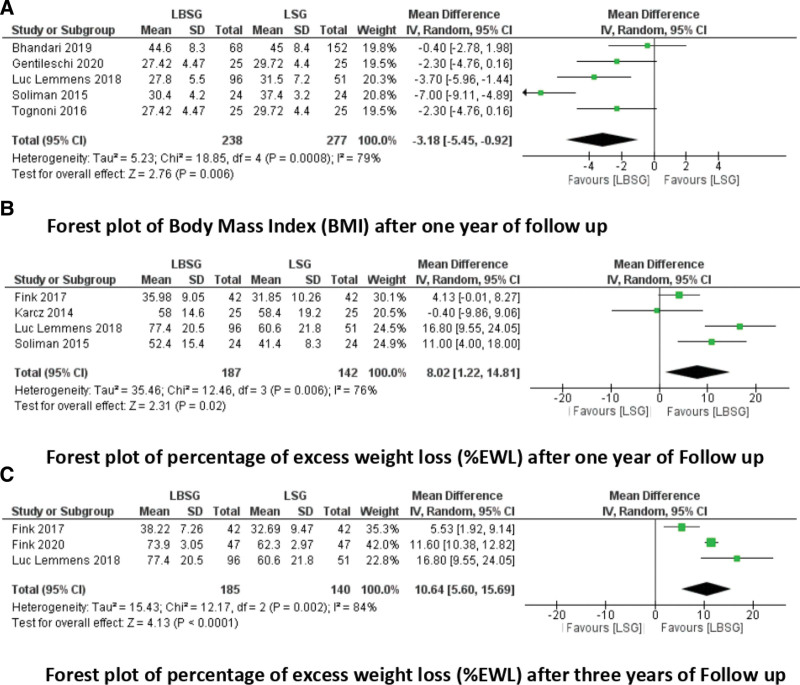
Forest plots of the anthropometric parameters.

BMI after 1 year: Five studies reported the BMI after 1 year of follow-up.^[17–19,22,23]^ They included 238 patients in the LBSG group and 277 in the LSG group (Fig. 2A). There was a lower BMI after 1 year of follow-up in the LBSG group than in the LSG group (MD = −3.18; 95% CI [−5.45, −0.92], *P* = .006), with low heterogeneity among the studies (Tau^2^ = 5.23 [*I*^2^ = 79%]).

%EWL after 1 year: Four studies reported the %EWL after 1 year of follow-up.^[16,18–20]^ They included 187 patients in the LBSG group and 142 patients in the LSG group (Fig. 2B). We observed a significantly greater %EWL in the LBSG group than in the LSG group (MD = 8.02; 95% CI [1.22, 14.81], *P* = .02).

%EWL after 3 years: Three studies reported the %EWL after 3 years of follow-up.^[19–21]^ They included 185 patients in the LBSG group and 140 in the LSG group (Fig. 2C). There was a greater %EWL in the LBSG group than that in the LSG group (MD = 10.60; 95% CI [5.60, 15.69], *P* < .001). There was moderate heterogeneity among the studies (Tau^2^ = 15.43 [*I*^2^ = 84%]).

5-years results: Long-term outcomes were reported in 2 studies.^[17,19]^ The %EWL values were reported by Lemmens et al^[19]^ and Bhandari et al.^[17]^ It was significantly greater in the LBSG group (86.7 ± 11.9 vs 57.8 ± 25, *P* = .003) and (90.9% vs 85.27%, *P* < .001), respectively. %EBMI was reported by Lemmens et al.^[19]^ It was significantly greater in the LBSG group (102.4 ± 19.3 vs 66 ± 32.8, *P* = .004).

### 3.3. Operative time

**Figure 3.**
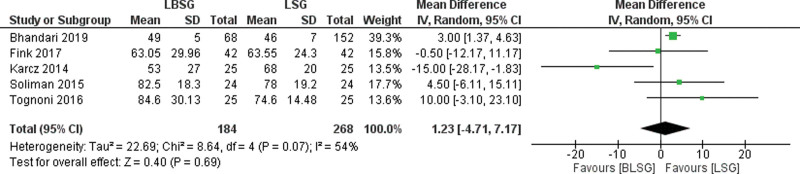
Forest plot of the operative time.

The operative time was reported in 5 studies.^[16–18,20,22]^ They included 184 and 268 patients in the LBSG and LSG groups, respectively (Fig. 3). In the pooled analysis, there were no differences between the 2 groups in terms of operative times (MD = 1.23; 95% CI [−4.71, 7.17], *P* = .69). There was moderate heterogeneity among the studies, with Tau^2^ = 22.69 (*I*^2^ = 54%).

### 3.4. Food intolerance

**Figure 4.**
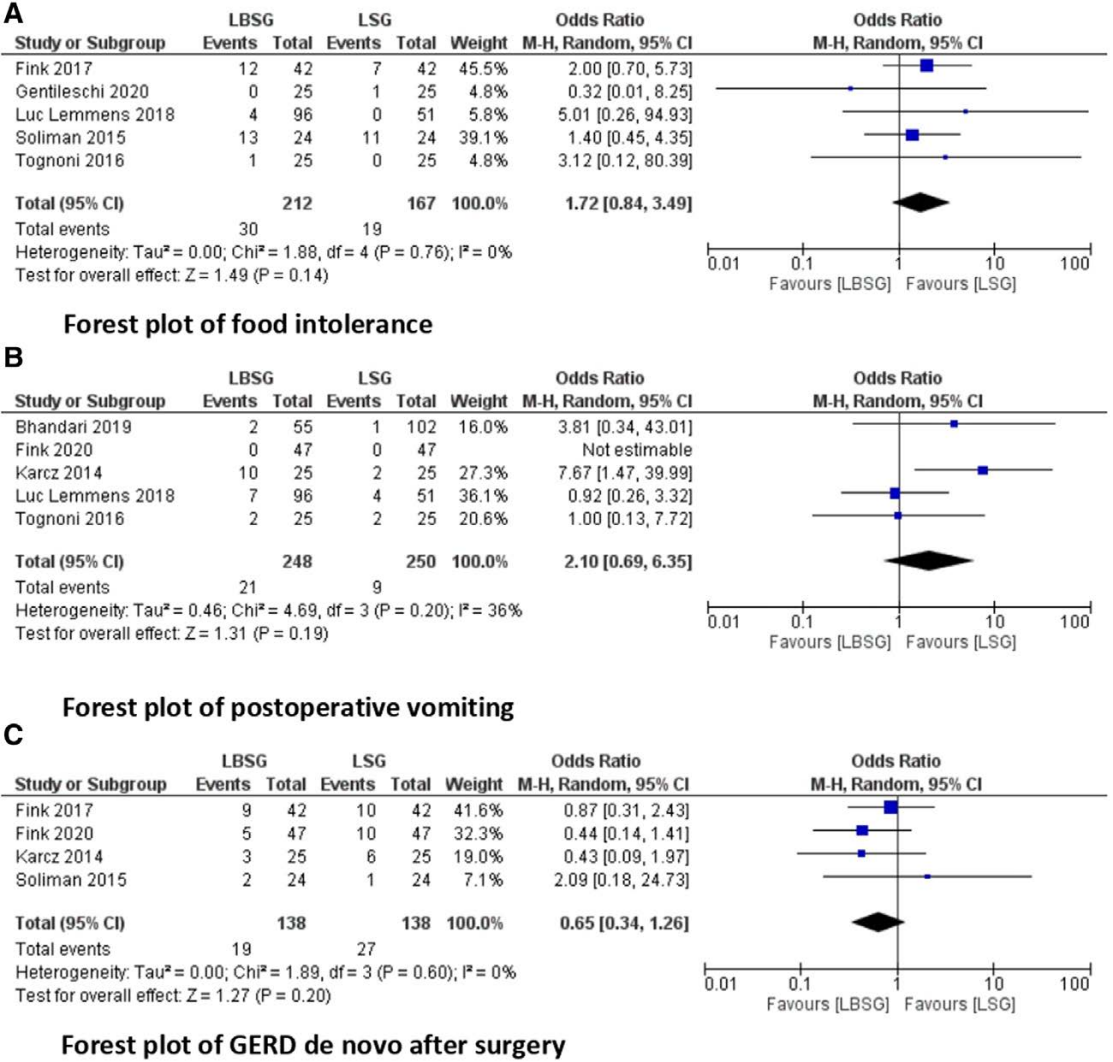
Forest plots of the postoperative outcomes.

Food intolerance was reported in 5 studies.^[18–20,22,23]^ It was reported in 30 of 212 patients in the LBSG group and in 19 of 167 patients in the LSG group (Fig. 4A). There was no significant difference between the 2 groups in terms of food intolerance (OR = 1.72; 95% CI [0.84, 3.49], *P* = .14).

### 3.5. Vomiting

Postoperative vomiting was reported in 4 studies.^[16,17,19,21,22]^ It was observed in 21 of 248 patients in the LBSG group and in out of 250 patients in the LSG group (Fig. 4B). There was no difference between the 2 groups with regard to vomiting rate (OR = 2.10; 95% CI [0.69, 6.35], *P* = .19).

### 3.6. De novo GERD

De novo GERD after surgery was reported in 4 studies.^[16,18,20,21]^ This outcome was observed in 19 of 138 patients in the LSBG group and in 27 of 138 patients in the LSG group (Fig. 4C). There was no difference between the 2 techniques in terms of the de novo GERD rate (OR = 0.65; 95% CI [0.34, 1.26], *P* = .2).

### 3.7. Postoperative complications requiring reoperation

Postoperative complications requiring reoperation are reported in Table 2. They were bleeding, stenosis, dysphagia, vomiting, band slippage, GERD, and weight regain.

**Table 2:** The different postoperative complications requiring reoperation.

First author	Complications	Management	
		LBSG group	LSG group
Bhandari	Inadequate weight loss or weight regain		Eleven converted to banded gastric bypasses, 6 converted 1-anastomosis gastric bypasses, and 2 re-sleeve).
Fink 2017	Band slippage	Band removal	
	GERD	Conversion to RYGBP for 2 patients	Conversion to RYGBP for 1 patient
	Bleeding	Revision due to staple line bleeding for 1 patient	Revision due to staple line bleeding for 1 patient
Gentileschi	Stenosis		Converted to RYGBP for 1 patient
Karcz	Vomiting	Band removal for 2 patients	
	Bleeding	Revision due to staple line bleeding for 1 patient	
Lemmens	Dysphagia	Band removal due to stenosis at the level of the ring	
Tognoni	Stenosis		Conversion to RYGBP for 1 patient

GERD = gastroesophageal reflux disease, LBSG = laparoscopic banded sleeve gastrectomy, LSG = laparoscopic sleeve gastrectomy, RYGBP = roux-en-Y gastric bypass.

### 3.8. Comorbidities resolution

The criteria for morbidity resolution were not standardized among the studies. Furthermore, a few studies have reported the percentage of patients with postoperative comorbidity resolution (Table 3). Therefore, we did not perform a pooled analysis for this variable of interest.

**Table 3:** The different preoperative comorbidities and comorbidities resolution.

First author	Preoperative comorbidities										Postoperative improvement of co-morbidities							
	Diabetes		Hypertension		Sleep apnea		GERD		Diabetes		Hypertension		Sleep apnea		GERD		GERD de novo	
	LBSG	LSG	LBSG	LSG	LBSG	LSG	LBSG	LSG	LBSG	LSG	LBSG	LSG	LBSG	LSG	LBSG	LSG	LBSG	LSG
Bhandari	15	31	25	69	2	7	–	–	–	–	–	–	–	–	–	–	–	–
Fink 2017	7	9	27	23	–	–	22	20	–	–	–	–	–	–	31	29	9	10
Fink 2020	11	6	26	20	–	–	11	6	1	2	6	9	–	–	7	2	5	10
Gentileschi	5	7	7	14	2	6	–	–	1	2	1	7	0	0	–	–	–	–
Karcz	6	6	12	15	–	–	9	6	–	–	–	–	–	–	12	12	3	6
Lemmens	12	12	22	17	32	13	–	–	–	–	–	–	–	–	–	–	–	–
Soliman	2	2	4	2	1	1	–	–	–	–	–	–	–	–	–	–	2	1
Tognoni	5	7	7	14	6	8	–	–	1	1	1	7	0	0	–	–	–	–

GERD = gastroesophageal reflux disease, LBSG = laparoscopic banded sleeve gastrectomy, LSG = laparoscopic sleeve gastrectomy.

### 3.9. Quality assessment of the included studies and reporting of the effects of banding SG

The ROBINS-I and RoB2 scores for the retained clinical trials are shown in Table 4. A summary of the evidence is presented in Table 5. This review shows that when LSG is banded, compared to non-banded LSG:

**Table 4:** Risk of bias assessment of the retained clinical trials.

Risk of bias 2 of the retained randomized controlled trials												
First author	Randomization process		Deviations from intended interventions		Bias in measurement of outcome		Bias to missing outcome data			Bias in selecting the reported results		Overall bias
Fink 2020	Low risk		High risk		Low risk		Low risk			Low risk		High risk
Gentileshi	High risk		High risk		Low risk		Low risk			Low risk		High risk
Tognoni	Some concerns		High risk		Low risk		Low risk			Low risk		High risk
ROBINS-I for risk of bias for non-randomized studies												
First author	Bias due to confounding	Bias in selection of participant into the study		Bias in classification of interventions		Bias due to deviations from intended interventions		Bias due to missing data	Bias in measurement of outcomes		Bias in selection of the reported result	Overall bias
Bhandari	Low risk	Low risk		Low risk		Low risk		Low risk	Serious risk		Low risk	Serious risk
Fink 2017	Low risk	Low risk		Low risk		Moderate risk		Moderate risk	Serious risk		Low risk	Serious risk
Karcz	Low risk	Low risk		Low risk		Low risk		Low risk	Serious risk		Low risk	Serious risk
Lemmens	Low risk	Low risk		Low risk		Low risk		Low risk	Serious risk		Low risk	Serious risk
Soliman	Low risk	Low risk		Low risk		Low risk		Low risk	Serious risk		Low risk	Serious risk

**Table 5:** The certainly of the evidence table.

Patient or population: Morbid obesity					
Setting: Intervention: LBSG; Comparison: LSG					
Outcomes	No of participants (studies) Follow-up	Certainty of the evidence (GRADE)	Relative effect (95% CI)	Anticipated absolute effects	
				Risk with LSG	Risk difference with LBSG
BMI after 1 yr	515 (2 RCTs + 3 CCTs)	⨁⨁◯◯Low†,‡	-	-	MD 3.18 kg/m^2^ lower (5.45 lower to 0.92 lower)
%EWL after 1 yr	329 (1 RCT + 3 CCTs)	⨁⨁◯◯Low†‡	-	-	MD 8.02 kg/m^2^ higher (1.22 higher to 14.81 higher)
%EWL after 3 yr	325 (1 RCT + 2 CCTs)	⨁⨁◯◯Low†,‡	-	-	MD 10.64 kg/m^2^ higher (5.6 higher to 15.69 higher)
Vomiting	341 (1 RCT + 4 CCTs)	⨁◯◯◯Very low†,‡	OR 1.90 (0.47–7.63)	54 per 1 000	44 more per 1000 (28 fewer to 250 more)
GERD de novo	276 (1 RCT + 3 CCTs)	⨁◯◯◯Very low†,‡	OR 0.65 (0.34–1.26)	196 per 1 000	59 fewer per 1000 (119 fewer to 39 more)
Intolerance to food	379 (3 RCTs + 2 CCTs)	⨁◯◯◯Very low†,‡	OR 1.72 (0.84–3.49)	114 per 1 000	67 more per 1000 (16 fewer to 196 more)
Operative time	452 (2 RCTs + 3 CCTs)	⨁◯◯◯Very low†,‡	-	-	MD 1.23 higher (4.71 lower to 7.17 higher)

GRADE Working Group grades of evidence. High certainty: we are very confident that the true effect lies close to that of the estimate of the effect. Moderate certainty: we are moderately confident in the effect estimate: the true effect is likely to be close to the estimate of the effect, but there is a possibility that it is substantially different. Low certainty: our confidence in the effect estimate is limited: the true effect may differ substantially from the effect estimate. Very low certainty: we have very little confidence in the effect estimate: the true effect is likely to be substantially different from the estimate of effect.

BMI = body mass index, CCT = controlled clinical trials, CI = confidence interval, GERD = gastroesophageal reflux disease, GRADE = Grading of Recommendations Assessment, Development, and Evaluation, LBSG = laparoscopic banded sleeve gastrectomy, LSG = laparoscopic sleeve gastrectomy, MD = mean difference, OR = odds ratio, RCT = randomized controlled trials.

*The risk in the intervention group (and its 95% confidence interval) is based on the assumed risk in the comparison group and the relative effect of the intervention (and its 95% CI).

† Small sample size is <400, suggesting issues with imprecision.

‡ *I*^2^ was >50% with a *P* value <.0001 suggesting substantial heterogeneity.

• It may ensure a lower BMI and %EWL after 1 year of follow-up with a reduction of 3.18 kg/m^2^ and 8.02 kg/m^2^ in the LBSG group and a probably significant reduction in %EWL after 3 years of follow-up.
• We do not know if it leads to additional vomiting, de novo GERD, intolerance to food, or longer operative time because the evidence is very uncertain.

## 4. Discussion

This systematic review with a meta-analysis of 753 patients comparing LBSG and LSG concluded that banding SG may ensure a lower BMI and %EWL after 1 year of follow-up and a significant reduction in %EWL after 1 year and 3 years of follow-up. There is no evidence to support LBSG in vomiting, de novo GERD, food intolerance, or operative time.

LSG is the most commonly performed procedure in the world. Its widespread use is due to its technical simplicity and fewer postoperative complications. However, long-term data have shown an alarming trend in weight regain.^[1,24]^ One of the most frequently reported factors leading to weight regain after LSG is remnant gastric tube dilation. For these reasons, many authors have suggested revisional surgery by performing a re-sleeve, conversion to a GBP, and 1 anastomosis duodenal switch.^[25]^ However, similar to sleeve failure, these procedures had some degree of overlap. The addition of a non-adjustable band around the gastric tube is widely advocated. Fobi et al^[26]^ initially described this technical modification in 1989, when a non-adjustable band was placed in a GBP.^[27]^ This technical variety leads to greater anthropometric results with an acceptable rate of band-related complications. For LBSG, the idea was that the band would hopefully increase both in magnitude and, more importantly, the durability of weight loss after these procedures without additional side effects or complications. Although this procedure has been performed for more than a decade, the prevalence of LBSG among all bariatric surgeries remains unknown. This systematic review and meta-analysis examined the risks and benefits of banding a SG.

Regarding the primary outcome concerning anthropometric parameters, short- and mid-term results of weight loss data 1 year and 3 years after surgery indicated higher weight loss in the LBSG group due to better weight stability.^[20,28]^ The pooled analysis concluded a lower BMI after 1 year in the LBSG with a low heterogeneity among the studies (Tau^2^ = 5.23 [*I*^2^ = 79%]) and greater %EWL with moderate heterogeneity among the studies (Tau^2^ = 15.43 [*I*^2^ = 84%]). The mean %EWL increased with the duration of follow-up, highlighting the efficacy of the band during this time. The non-standardized surgical procedures could explain the heterogeneity among the studies: the distance from the pylorus to the start of sleeve formation, the size of the gastric calibration tube, the type of gastric band used, ring circumference, and placement from the gastroesophageal junction. However, these studies employed 35 to 38 French tubes for calibration, and according to the available data, there is no clear link between gastric tube size and anthropometric results. On the other hand, higher postoperative gastric remnant tube volume and neofundus formation have been linked to deteriorating weight loss.^[29,30]^ In addition, the distance between the band and gastroesophageal junction varied slightly, ranging from 4 to 6 cm. The small sample size of the included patients made it difficult to determine the best band placement position. Currently, there are no comparative studies comparing the different band placements.

Various band devices, such as the GaBP Ring Autolock, double-layer mesh (Gortex mesh), and MiniMizer ring, have also been employed by different researchers. Various reports in the literature make it difficult to determine whether 1 band has an edge over the other. The MiniMizer ring appears to have an advantage over other rings in terms of ease of implantation, allowing for easy modification of the desired diameter, and producing a pseudo capsule that allows for easy removal.^[31]^ Soliman et al^[18]^ examined the pictures published in their article and approximated the mesh. Therefore, the resulting length of the mesh must be shorter than 6.5 to 7 cm compared with a 6.5 to 7 cm silicone ring. This study could also explain the heterogeneity after pooling the different results.

Regarding 5-years outcomes, only 2 studies^[17,19]^ investigated the data using different measures. Therefore, this meta-analysis was not feasible. The %EWL was significantly greater in the LBSG group in the studies by Lemmens et al^[19]^ and Bhandari et al,^[17]^ with a greater %EBMI in the LBSG group according to Lemmens et al.^[19]^ The exact mechanism underlying greater anthropometric results in the LBSG group remains unclear. This could be explained by the continuous weight gain observed in the Non-banded SG group. Dilation of the remnant gastric tube may be one of the reasons for this. A comparison of computed tomography sleeve volumetry should prove this assumption between LBSG and LSG. Furthermore, the band around the sleeve can reduce appetite and enhance satiety via the perigastric neural pathway.^[19,20]^ One of the biggest advantages of LBSG is the lesser amount of weight regain, not by the restriction effect but by preventing dilatation.

Our study showed that band placement did not significantly increase the operative time. The operative time was 1.23 minutes longer in the LBSG group than in the LSG group. Moderate heterogeneity was observed among the studies. The absence of standardized criteria to measure operative time could explain this heterogeneity in the appreciation of operative time. The time-to-band setup varied according to the different band types.

GERD in obese patients presents a well-known health problem with an increased risk of Barrett esophagus and cardiac adenocarcinoma.^[32]^ This is a well-discussed topic after the LSG.^[33]^ Several studies had assessed surgical tools to reduce the post operative GERD.^[34,35]^ In our review, we found no difference in the de novo GERD rate between the LBSG and LSG groups. This mostly agrees with the findings of the included studies.^[16,18,20,21]^ However, in these studies, the hazardous evolution of GERD after surgery: worsening or improvement should be mentioned. In addition, there is insufficient data concerning the use of proton pump inhibitors. A purely clinical assessment without any evaluation of the GERD questionnaire or quality of life was considered, which should be encouraged in future studies. In contrast, the findings in our report led to the hypothesis that the gastric band after SG does not increase GERD symptoms.

Regarding the high rate of complications and failure after the gastric band, the major concern with LBSG is the risk of introducing new problems with the band and reoperation in some cases. This concept keeps the surgeon away from the bands. However, the concept of LBSG differs from that of the gastric band, with an absence of absolute and constant pressure on the gastric wall, which causes some problems. In a systematic review and meta-analysis, Buchwald et al^[36]^ included 8707 patients reporting a band erosion rate of 2.3% and band slippage rate of 1.5%. The authors concluded that band-related complications may not occur frequently. Moreover, the incidence of postoperative complications requiring reoperation was not higher in the LBSG group. Our study did not find a difference between the 2 groups regarding food intolerance and postoperative vomiting. In addition, the authors did not mention whether clinical food intolerance was proven radiologically to relate it to the band. This outcome had to be evaluated more precisely in the long-term. In conclusion, the rate of band complication after LBSG is very low.

This could be a mature concept of making the bands looser, not quite as tight, with a circumference of a minimum of 6.5 to 7 cm. The real purpose of the band in gastric banding is to prevent major stretching that tends to occur over time. Lemmens et al^[19]^ reported upper gastrointestinal symptoms when a 605 cm band was used. These symptoms improved with the 7.5 cm band. Fink et al^[21]^ increased the band closure circumference to 7.5 cm after their first published study.^[20]^ Everything work in the first year, LSG, and even the band can work relatively well, but a good procedure is effective years after surgery. This explains the lower weight regains in the LBSG group and the more significant weight loss at 3 and 5 years of follow-up.

The other major issue with this band is erosion. This complication was reported in only 1 patient in this review. This is due to the erosion rate of the band material and its placement at 4 to 6 cm from the gastroesophageal junction. In addition, erosion is rare and can be managed endoscopically. Thus, we believe that these new emerging data could be used for LBSG.

However, one of the embarrassing aspects to mention in the case of LBSG is the management of postoperative fistulas. We should always perform laparoscopy, drain the fluid collection, remove the ring most of the time, and place an esophageal stent to cover the fistula. This leakage can cause band erosion.

Regarding comorbidity resolution, the data were not standardized among the studies. Most of the retained studies had variable follow-up periods and did not assess this outcome. Thus, pooled analysis was not applicable. We report the available data presented in the literature.

Our study compared the safety and efficacy of LBSG and LSG. This study has several limitations. Owing to the reduced number of RCTs, lack of some outcomes, and lack of long-term follow-up, we included additional CCTs with a risk of selection bias and low to moderate heterogeneity in the 2 outcomes, highlighting the need for additional RCTs on this topic. In addition, the follow-up period was limited to 5 years, and the onset of band-related complications such as perforation, migration, or erosion could take longer to appear. Furthermore, the most appropriate method of reporting weight loss after bariatric surgery^[37]^ is an issue regarding the outcome measures used in the retained studies. For example, the authors used 4 different outcome measures in their anthropometric data: BMI, excess weight loss, excess BMI loss, and BMI loss, making this comparison difficult. Therefore, our findings should be cautiously interpreted. We should wait for more long-term data, because we did not consider the LSG rate. This is fine in these studies and does not require any intervention or revision. Probably, 30% to 40% presented weight regain.^[38]^

We did not assess why they had to regain weight (eating habits, behaviors, physical activities, etc). If these patients require more aggressive surgery, the band is previously placed like a SADI-S, re-sleeve, duodenal switch, or conversion to gastric bypass. About 50% of LSG did not present weight regain, and not every sleeve failed because they dilated, highlighting an over-or incorrect treatment by banding them from the beginning.^[38]^ Salvi et al^[39]^ retrospectively compared LBSG with one-anastomosis gastric bypass for the treatment of obesity. They concluded that both operations produced excellent weight loss and short to intermediate term maintenance. There was better resolution of comorbidities after one-anastomosis gastric bypass at the expense of a higher incidence of nutrient deficiency and some protein caloric malnutrition. However, further prospective and larger studies are required to confirm these findings.

In conclusion, this review shows that LBSG may ensure a lower BMI and %EWL after 1 year of follow-up, with a large reduction in %EWL after 3 years in the LBSG group. However, we do not know if it leads to additional vomiting, de novo GERD, intolerance to food, longer operative time, complications requiring reoperation, and comorbidity remission because the evidence is very uncertain. Additional RCTs with larger sample sizes and longer follow-ups are mandatory for a greater placement of LBSG in the therapeutic armature of morbid obesity to investigate which patients need to undergo LBSG since the first procedure.

## Acknowledgments

The authors acknowledge the help of Hanene Chaouch, Pharmacist (Monastir University of Pharmacy, Monastir University), for the extraction and analysis of data.

## Author contributions

**Conceptualization:** Hani Oweira.

**Data curation:** Wah Yang, Amine Gouader, Krimi Bassem, Guillaume Pourcher, Hani Oweira.

**Formal analysis:** Wah Yang, Amine Gouader.

**Funding acquisition:** Amine Gouader, Adriano Carneiro da Costa, Hani Oweira.

**Investigation:** Wah Yang, Amine Gouader, Krimi Bassem.

**Methodology:** Mohamed Ali Chaouch, Amine Gouader, Krimi Bassem.

**Project administration:** Krimi Bassem.

**Resources:** Mohamed Ali Chaouch, Krimi Bassem, Guillaume Pourcher.

**Software:** Mohamed Ali Chaouch, Krimi Bassem.

**Supervision:** Wah Yang, Adriano Carneiro da Costa.

**Validation:** Mohamed Ali Chaouch, Wah Yang, Adriano Carneiro da Costa, Hani Oweira.

**Visualization:** Adriano Carneiro da Costa, Guillaume Pourcher, Hani Oweira.

**Writing – original draft:** Mohamed Ali Chaouch, Adriano Carneiro da Costa.

**Writing – review & editing:** Guillaume Pourcher, Hani Oweira.
